# T cell activation and anti-tumor efficacy of anti-LAG-3 antibodies is independent of LAG-3 – MHCII blocking capacity

**DOI:** 10.1186/2051-1426-3-S2-P183

**Published:** 2015-11-04

**Authors:** Saso Cemerski, Shuxia Zhao, Melissa Chenard, Jason Laskey, Long Cui, Rinkan Shukla, Brian Haines, Edward Hsieh, Maribel Beaumont, Jeanine Mattson, Wendy Blumenschein, Heather Hirsch, Laurence Fayadat-Dilman, Linda Liang, Rene De Waal Malefyt

**Affiliations:** 1 Merck Research Laboratories, Boston, MA, USA; 2 Merck Research Laboratories, Palo Alto, CA, USA

## 

LAG-3 has been shown to act as an inhibitory molecule involved in the regulation of T cell activation, proliferation and homeostasis. Exhausted T cell populations that evolve in the tumor microenvironment or during chronic viral infections show coordinated expression of LAG-3 and PD-1. LAG-3 is structurally related to CD4 and binds to MHCII. Anti-LAG-3 antibodies have demonstrated preclinical efficacy in several disease models in particular when combined with anti-PD-1 antibodies. Studies have proposed that LAG-3 blockade is efficacious in both CD4^+^ and CD8^+^ T cells despite the lack of significant MHCII levels on CD8^+^ T cell. In the present study, we evaluated if anti-LAG-3 efficacy is dependent on the ability of the antibody to inhibit the binding of LAG-3 to MHCII.

We have compared in a series of *in vitro* assays the biological activity of two distinct anti-mouse LAG-3 antibodies: C9B7W which does not block the LAG-3 – MHCII interaction and an in-house generated antibody 28G10 that strongly blocks the interaction between LAG-3 and MHCII. Biophysical characterization revealed that C9B7W and 28G10 recognize distinct epitopes on LAG-3, therefore explaining the difference in LAG3-MHCII interruption. However, no differences were observed in T cell activation assays between the two antibodies using TCR transgenic CD4^+^ T cells. In addition, their ability to synergize with an anti-PD-1 antibody was also comparable.

To understand if the overall enhancement in CD4^+^ T cell activation by C9B7W and 28G10 was achieved through different mechanisms, we profiled gene expression in T cells stimulated in the presence of anti-LAG-3 antibodies. No significant difference was found between the two antibodies. Consistent with this observation, we did not see an additive effect of C9B7W and 28G10 when used together *in vitro*. Neither antibody demonstrated a significant effect on the activity of TCR transgenic CD8^+^ T cells in *in vitro* functional assays.

Furthermore, the two antibodies demonstrated comparable anti-tumor efficacy in *in vivo* syngeneic tumor models when dosed in combination with anti-PD-1.

In conclusion, our studies demonstrate that the activity of LAG-3-targeting antibodies is not associated with their ability to disrupt LAG-3-MHCII interaction. This would suggest that anti-LAG-3 antibodies should enhance both CD4^+^ and CD8^+^ T cell function. Case in contrast, our *in vitro* data demonstrates that LAG-3 targeting augments the activation of CD4^+^ T cells significantly more than CD8^+^ T cells.

